# Alkaline-Earth Metals-Doped Pyrochlore Gd_2_Zr_2_O_7_ as Oxygen Conductors for Improved NO_2_ Sensing Performance

**DOI:** 10.1038/s41598-017-04920-1

**Published:** 2017-07-05

**Authors:** Fulan Zhong, Jiwu Zhao, Lanqian Shi, Yihong Xiao, Guohui Cai, Yong Zheng, Jinlin Long

**Affiliations:** 1 National Engineering Research Center of Chemical Fertilizer Catalyst (NERC-CFC), School of Chemical Engineering, Fuzhou University, No. 523 Gongye Road, Fuzhou, 350002 Fujian P.R. China; 2 State Key Laboratory of Photocatalysis on Energy and Environment, School of Chemistry, Fuzhou University, Fuzhou, 350116 P.R. China

## Abstract

This work proposed a novel strategy to fabricate highly-stable NO_2_ sensor based on a pyrochlore-phase Gd_2_Zr_2_O_7_ oxygen conductor. The incorporation of alkaline-earth metals distinctly enhances the sensing performance of the Gd_2_Zr_2_O_7_ based sensors. The excellent sensor based on Gd_1.95_Ca_0.05_Zr_2_O_7+δ_ exhibits rapid response-recovery characteristics with the maximum response current value (ΔI = 6.4 μA), extremely short 90% responce (3 s) and 90% recovery (35 s) time towards 500 ppm NO_2_ at 500 °C, which is better than that of commercial YSZ under the same condition. The ΔI value towards NO_2_ is much higher than those towards other gases (CH_4_, C_3_H_6_, C_3_H_8_, CO, NO, SO_2_, C_2_H_4_, CO_2_ and C_2_H_6_), exhibiting excellent selectivity for detecting NO_2_. The response signal basically maintains a stable value of 6.4 μA after the sensors was stored for half a month and a month. The outstanding selectivity and highly stability of the NO_*2*_ sensors based on Gd_2−x_M_x_Zr_2_O_7+*δ*_ are expected to a promising application in automotive vehicles.

## Introduction

Nitrogen oxides (NO_x_, NO and NO_2_), which are mainly released from automotive engines, are harmful to humans and the environment^1–3^. To monitor NO_2_ emission, great efforts are devoted to develop high performance and compact solid electrolyte type NO_2_ sensor with a sensitive, stable, selective and quick response^4–11^. As to NO_2_ sensors, the solid electrolytes play a very important role in the sensing performances. To further improve the properties of the NO_2_ sensors, great efforts have been devoted to improve the ionic conductivity of solid electrolytes and develop novel oxygen conductors^12–14^. YSZ possesses exceedingly high ionic conductivity only when the temperature exceeds 1073 K, whereas the high operating temperature inevitably not only limits the selection of compatible electrode and interconnect materials, but shortens the service life of the sensor^12–14^. Perovskite-phase (ABO_3_) solid electrolytes have been recently indicated to be quite optimal materials for NO_2_ sensors. One of the most promising materials is doped LaGaO_3_^15^, such as (La, Nd)_0.8_Sr_0.2_Ga_0.8_Mg_0.2_O_2.8_^16, 17^, the conductivity of which is comparable to YSZ. Unfortunately, Gallium has a volatility, which greatly restricts the application of such sensor in NO_2_ detection too. Inspired by the studies on doped LaGaO_3_ above, we have recently reported the NO_2_ sensors based on perovskite-phase GdAlO_3_ substrates, in where Ca was arranged at A-sites^18^. However, the response and recovery time is very long in excess of 119 and 92 s, respectively. The most key reason leading to the long response and recovery time can be related to the oxygen transport capacity of the solid electrolyte and the ability to capture NO_2_ of the sensor^19^. Therefore, it stimulates us to seek a novel solid electrolyte material, expecting to further enhance oxygen vacancies and NO_2_ adsorption capacity to improve the electrochemical catalytic performance.

For the general amperometric NO_2_ sensor, NO_2_ gas is first absorbed on the porous surface of the sensing electrode (SE), which makes NO_2_ gas many contacts with the surface of the SE grains that is high catalytic activity, making NO_2_ gas decompose into NO and NO further decompose into N_2_ with Eqs 1 and 2 in the vicinity of the electrode, respectively^20, 21^. The decomposition substance will diffuse through the bulk electrode which makes the target gas NO_2_ hardly reach the electrode/electrolyte interface, causing a low sensitivity towards NO_2_. The higher catalytic activity of NO_2_ gas decomposition into NO or N_2_ gives the lower sensitivity towards NO_2_. Therefore, the sensitivity of the sensor strongly depends on the catalytic activity of the oxide electrode. Recently, p-type semiconducting metal-oxides have drawn a lot of attention as sensing electrode, such as NiO, TeO_2_, Co_3_O_4_ and CuO. Among these oxides, NiO is widely used as sensing electrode of NO_2_ sensor due to its non-poisonous and large NO_2_ adsorption capacity. To improve the sensitivity of NiO-based sensor, conventional tactics is to reduce the catalytic activity of the oxide electrode via adding the corresponding electrolyte material such as YSZ to NiO in order to extend the length of three phase boundary (TPB), which will necessarily influence the adsorption capacity of NO_2_. As it is well-known, the larger the capacity of NO_2_ adsorption is, the faster the cathodic reaction rate of Eq. 3 is as well as the higher the sensitivity of the sensor is. A fraction of NO generated by Eq. 3 would be further reduced to N_2_ by gaining electrons (Eq. 4) due to the high catalytic activity of SE, which in turn promotes the generation rate of O^2−^ on SE. The O^2−^ generated by the cathodic reaction is quickly transported along the direction of the electrolyte grains to the reference electrode (RE), where the anodic reaction (Eq. 5) takes place. In whole of the electrochemical reaction cycle, the rate-determining step is strongly dependent on the oxygen ion carriers to modulate the oxygen transport capacity of the solid electrolyte, the adsorption–desorption behavior of NO_2_ at the electrolyte/electrode interface. How to restrain NO_2_ gas catalytic decomposition on SE and enhance oxygen vacancies and NO_2_ adsorption capacity is one of key problems for the fabrication of the NO_2_ sensors.

1 $${\rm{Decomposition}}\,{\rm{reaction}}:{{\rm{NO}}}_{2}\to {\rm{NO}}+1/2{{\rm{O}}}_{2}$$

2 $${\rm{NO}}\to 1/2{{\rm{N}}}_{2}+1/2{{\rm{O}}}_{2}$$

3 $${\rm{Cathodic}}\,{\rm{reaction}}:{{\rm{NO}}}_{2}+2{{\rm{e}}}^{-}\to {\rm{NO}}+{{\rm{O}}}^{2-}$$

4 $${\rm{NO}}+2{{\rm{e}}}^{-}\to 1/2{{\rm{N}}}_{2}+{{\rm{O}}}^{2-}$$

5 $${\rm{Anodic}}\,{\rm{reaction}}:{{\rm{O}}}^{2-}\to 1/2\,{{\rm{O}}}_{2}+2{{\rm{e}}}^{-}$$

Generally, the function of the solid electrolyte with high conductivity used for the sensor is to only transport oxygen ion as the medium. Expectedly, there exists in a solid electrolyte with high concentration of oxygen vacancies that can not only carry the oxygen ion but simultaneously modulate NO_2_ transport capacity at mild-temperature. Compared to the perovskite-phase binary oxides (ABO_3_), pyrochlore-phase oxides with the general formula of A_2_B_2_O_7_□, where six oxygen sites are always fully occupied while the seventh can be arranged in an additional oxygen non-stoichiometry “□”, exhibit very high intrinsic concentration of oxygen vacancies with the minimal number of 12.5%^22, 23^. Interestingly, introducing disordered extra vacancies can further enhance the conductivity of materials. For example, Ca-doped Gd_2_Ti_2_O_7_ (Gd_1.9_Ca_0.1_Ti_2_O_6.95_), the ion conductivity is as high as 0.05 S cm^−1^ at 800 °C over a large p_O2_ range (10^−1^ to 10^−20^ atm)^22^. Another advantage of the pyrochlore-phase oxides (A_2_B_2_O_7_□) can provide both A sites for doping cations with larger ionic radius and B sites with smaller ionic radius to adjust the range of 1.46 ≤ r (A^3+^)/r (B^4+^) ≤1.78 that is the prerequisite to form stable pyrochlore structure^24^, making them promise hosts for solid electrolytes for NO_2_ sensor in the intermediate-temperature^25^.

In the family of pyrochlore-phase compounds, it was reported that Gd_2_Zr_2_O_7_ exhibited the highest ionic conductivity (1 × 10^−3^ S cm^−1^) at the intermediate temperature of 1000 K^26^. Several studies demonstrated that the incorporation of Ti cations at B sites and Nd cations at A sites resulted in the enhanced conductivity of pyrochlore-phase Gd_2_Zr_2_O_7_ in the temperature range of 773–973 K^27, 28^. In this work, we studied firstly the incorporation of alkaline earth metals (Ca, Sr, and Ba) in pyrochlore-phase Gd_2_Zr_2_O_7_ based on the following two core considerations: (1) Incorporation of alkaline earth metals creates more amounts of oxygen vacancy into the solid electrolyte and increases oxygen migration to facilitate the anodic reaction; (2) Alkaline earth metals serves as a dopant in view of its strong NO_2_ storage capacity required for the electrochemical catalytic performance at low and moderate temperatures^29–32^, which is in favor of the enrichment of NO_2_ at the interface between SE and solid electrolyte, consequently active for NO_2_ sensing. And then we fabricated several amperometric-type NO_2_ sensors based on the alkaline earth metals doped pyrochlore Gd_2_Zr_2_O_7_ oxygen conductors with NiO as the SE and a noble metal Pt as the RE. The results showed that the incorporation of alkaline earth metals distinctly enhanced the conductivity of Gd_2_Zr_2_O_7_, and the highest conductivity reached up to 9.81 × 10^−3^ S cm^−1^ at 1173 K. The optimal NO_2_ sensor based on the Gd_1.95_Ca_0.05_Zr_2_O_7+*δ*_ oxygen conductor showed the highest response current value, the shortest response and recovery time at 500 °C, which is better than that of the sensor based on commercial YSZ with NiO SE material. The outstanding selectivity and highly stability of the NO_*2*_ sensors based on Gd_2−x_M_x_Zr_2_O_7+*δ*_ showed a promising application in automotive vehicles.

## Results and Discussion

**Figure 1:**
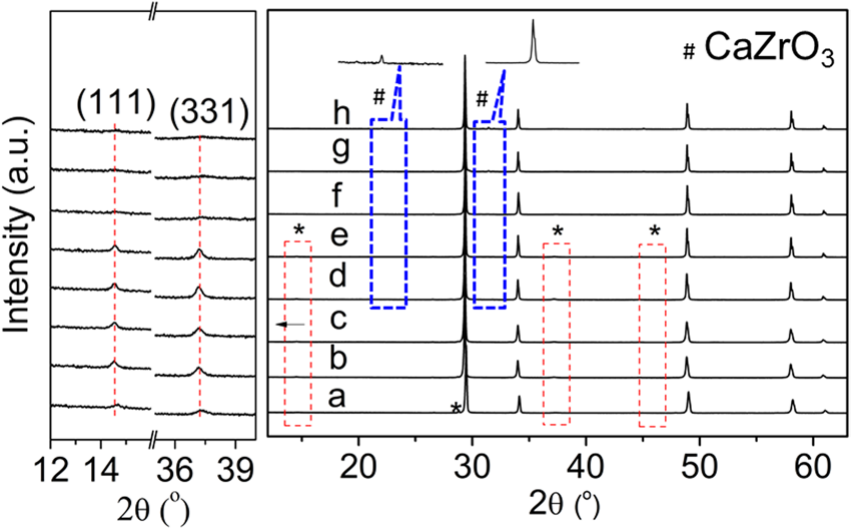
XRD patterns of Gd_2−x_Ca_x_Zr_2_O_7+*δ*_ powders calcined at 1500 °C for 4 h: (a) x = 0, (b) x = 0.02, (c) x = 0.05, (d) x = 0.1, (e) x = 0.15, (f) x = 0.2, (g) x = 0.25, (h) x = 0.3. Left insert shows an enlargement of some areas of the XRD patterns. The symbol “*” represents the superstructure peaks.

XRD patterns of Gd_2−x_Ca_x_Zr_2_O_7+δ_ samples calcined at 1500 °C for 4 h in air are presented in Fig. 1. It is observed that pure Gd_2_Zr_2_O_7_ exhibits an ordered pyrochlore-phase structure, which is characterized by the presence of the typical superstructure diffraction peaks at 2θ ≈ 14° (111), 28° (311), 37° (331) and 45° (511)^33–35^. As seen from Fig. 1, with the substitution of Gd^3+^ cations by Ca^2+^ cations, Gd_2−x_Ca_x_Zr_2_O_7+δ_ (0 < x < 0.2) can maintain the pyrochlore-phase structure due to the existence of the superstructure peaks. However, the pyrochlore superstructure reflections lost and the samples display a defective fluorite-phase structure with x ≥ 0.2. This means that the phase transition from pyrochlore to defect fluorite happens when the doping content x is beyond 0.2. Interestingly, perovskite structure CaZrO_3_ will not produce until x ≥ 0.1. Figure S1 represents the XRD patterns of Gd_2−x_Sr_x_Zr_2_O_7+δ_ and Gd_2−x_Ba_x_Zr_2_O_7+δ_ for the compositions corresponding to x = 0–0.2, whereas they exhibit the onsets of phase separation to pyrochlore [ICDD PDF 16–0799], perovskite structure SrZrO_3_ [ICDD PDF 74–2231], and BaZrO_3_ [ICDD PDF 89–2486] (marked by asterisks) even when the doping concentration is very low such as x = 0.02. This is attributed to the great difference of the ionic radius of Gd^3+^ and Sr^2+^ (Ba^2+^), resulting in the difficult substitution of small Gd^3+^ by large Sr^2+^ or Ba^2+^ cations. In whole of doping concentration, Gd_2−x_Sr_x_Zr_2_O_7+δ_ and Gd_2−x_Ba_x_Zr_2_O_7+δ_ retain the pyrochlore-phase structure.

**Figure 2:**
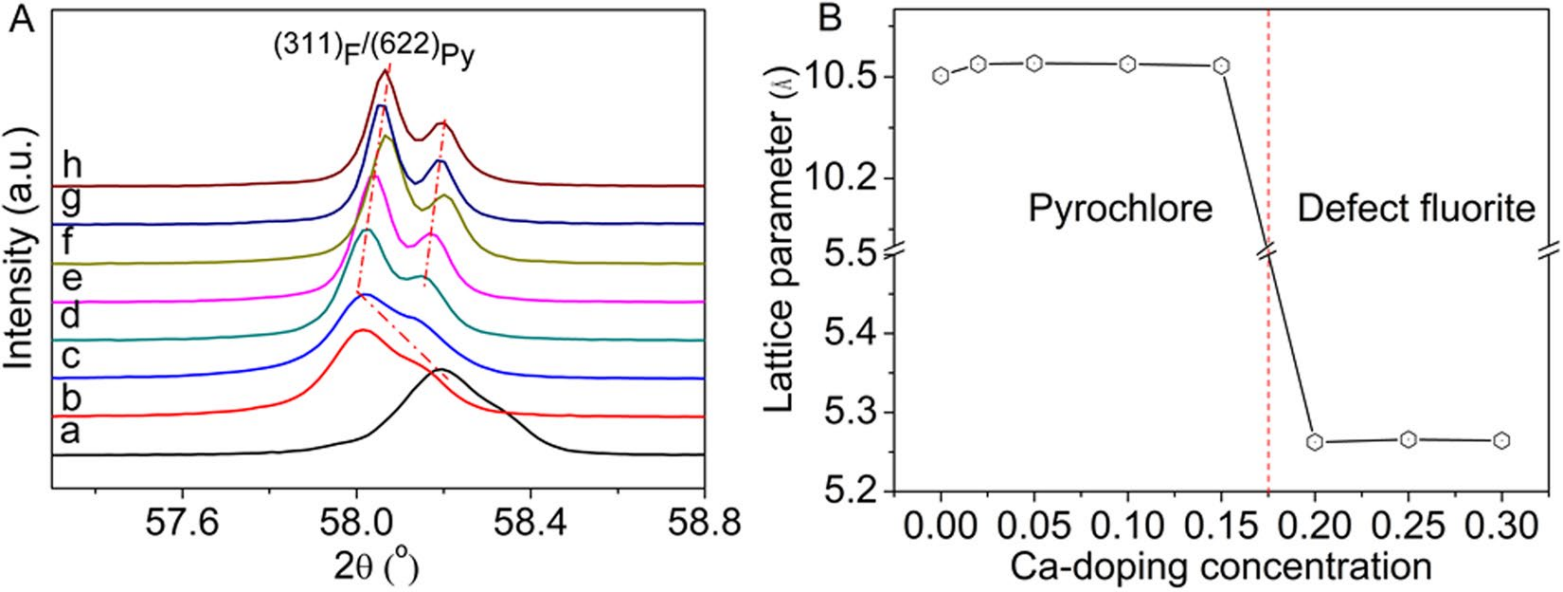
(**A**) (311)_F_/(622)_Py_ peak of Gd_2−x_Ca_x_Zr_2_O_7+δ_ samples in 2θ range of 56.5–57.5°:(a) x = 0, (b) x = 0.02, (c) x = 0.05, (d) x = 0.1, (e) x = 0.15, (f) x = 0.2, (g) x = 0.25, (h) x = 0.3; (**B**) Lattice parameters of Gd_2−x_Ca_x_Zr_2_O_7+*δ*_ in different phase regions.

It is noted from Fig. 2A that the peak of (311)_F_/(622)_Py_ for Gd_2−x_Ca_x_Zr_2_O_7-δ_ distinctly shifts towards lower angle for x ≤ 0.05, and then tardily shifts towards higher angle for x ≥ 0.1, predicating the lattice expansion as Ca^2+^ is introduced, which probably induces variation in oxygen vacancies. The cell parameters of all the compositions of Gd_2−x_Ca_x_Zr_2_O_7-δ_ samples were calculated using MDI Jade program, and the results were depicted in Fig. 2B. Clearly, the cubic lattice parameters with pyrochlore-phase structure display a rapid increase for x ≤ 0.05, and then gradual decrease for x ≥ 0.1, whereas the lattice parameters of the samples with defect fluorite structure are nearly half of the corresponding pyrochlore value. Since Ca^2+^ possesses similar ionic radius to Gd^3+^ other than Zr^4+^ ^36–38^, the excess Ca^2+^ tends to be arranged in A-site. The substitution of Gd^3+^ by a fraction of Ca^2+^ is favorable to the pyrochlore-phase structure as the ionic radius of Ca^2+^ is slightly larger than that of Gd^3+^ ^36, 37^, which makes the ionic radius ratio of r(Gd^3+^-Ca^2+^)_average_/r(Zr^4+^) larger than 1.46 and inevitably creates larger A-site volume. However, Ca^2+^ can only substitute for a fraction of Gd^3+^ because the extent of lattice distortion of pyrochlore structure is limited. Too much Ca can combine with Zr at B-site to form perovskite structure CaZrO_3_, resulting in the phase transition from pyrochlore to defect fluorite structure. The split and shift of the peak of (311)_F_/(622)_Py_ towards higher angle for Gd_2−x_Ca_x_Zr_2_O_7-δ_ (x ≥ 0.1) in Fig. 2A are attributed to the lattice disordering and the phase change of the formation of a new matter CaZrO_3_ due to the dissociation of doped Ca ions from the Gd_2_Zr_2_O_7_ lattice structure.

**Figure 3:**
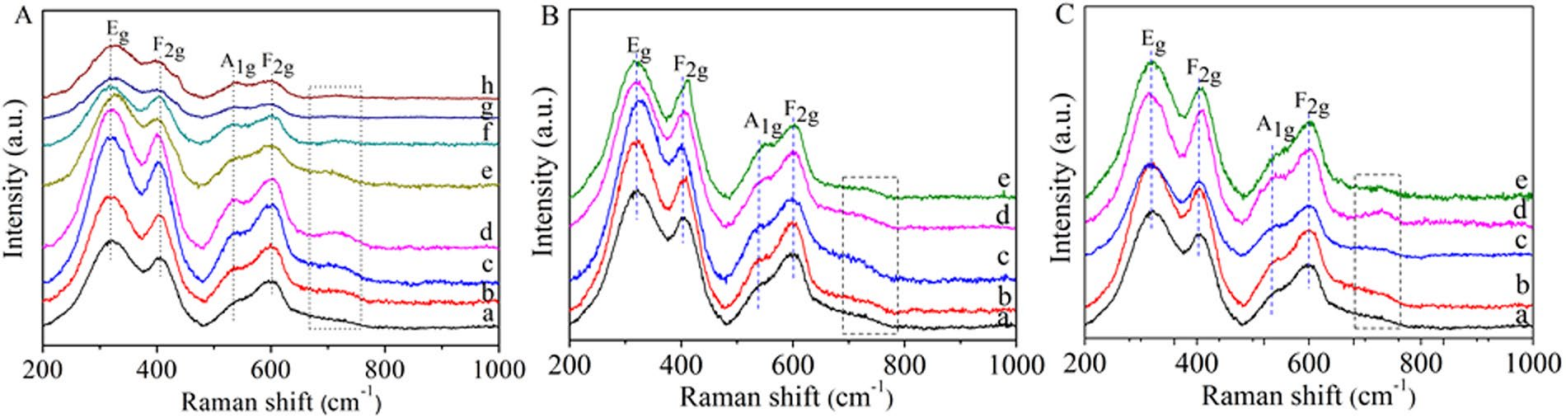
Raman spectra of Gd_2−x_Ca_x_Zr_2_O_7+*δ*_ (**A**), Gd_2−x_Sr_x_Zr_2_O_7+*δ*_ (**B**), and Gd_2−x_Ba_x_Zr_2_O_7+*δ*_ (**C**) powders calcinated at 1500 °C for 4 h: (a) x = 0, (b) x = 0.02, (c) x = 0.05, (d) x = 0.1, (e) x = 0.15, (f) x = 0.2, (g) x = 0.25, (h) x = 0.3.

To further confirm the structure, Raman spectroscopic investigations were carried out on all these samples at ambient conditions in the range 200–1000 cm^−1^, as shown in Fig. 3. Group theoretic alanalysis for the defect pyrochlore-phase compounds with space group Fd3m predicts six-Raman active modes, which are A_1g_ + E_g_ + 4F_2g_^39^. The spectrum of the Gd_2_Zr_2_O_7_ pyrochlore only shows four distinct bands due to the disorder, which is in quite good agreement with the literature^40^. A very obvious band at ~300 cm^−1^ that is identified as the E_g_ mode has been observed, whereas the other two vibrational frequencies at 412 and 608 cm^−1^ may be assigned to two of the four F_2g_ modes. The Raman-active band at 518 cm^−1^ has been assigned as the A_1g_ mode. As seen from Fig. 3A, the Raman spectra of Gd_2−x_Ca_x_Zr_2_O_7-δ_ (0.2 < x ≤ 0.3) is reduced to a broad continuum of density of states. It has been reported that the Raman spectra of the defect fluorites (A_0.5_B_0.5_O_1.75_) has a single broad band as the seven oxygen ions in the fluorite structure are randomly distributed over the eight anion sites^40^. This implies that the phase transition from pyrochlore to defect fluorite has happened for Gd_2−x_Ca_x_Zr_2_O_7-δ_ (0.2 < x ≤ 0.3) samples, which is agreement with the XRD result above. In addition, a new band is observed at around 720 cm^−1^ when M is introduced at the A site of the lattice. This new vibrational mode is assigned to the M–O symmetrical stretch vibration. These results further confirm that M is introduced to the A site of the lattice.

**Figure 4:**
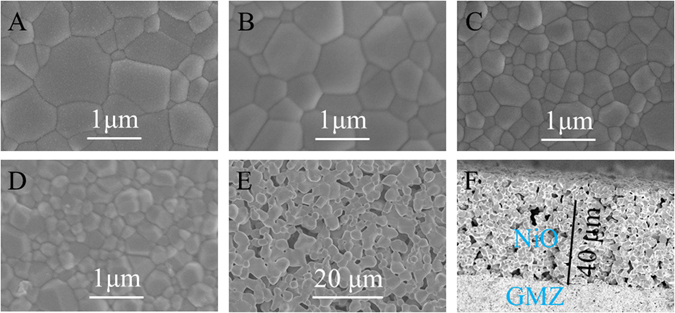
SEM images of the top-view of (**A**) Gd_2_Zr_2_O_7_, (**B**) Gd_1.95_Ca_0.05_Zr_2_O_7+*δ*_, (**C**) Gd_1.95_Sr_0.05_Zr_2_O_7+*δ*_, (**D**) Gd_1.95_Ba_0.05_Zr_2_O_7+*δ*_ substrates calcined at 1500 °C for 4 h; (**E**) the surface of NiO electrode; (**F**) cross-section image for the NiO porous layer.

**Figure 5:**
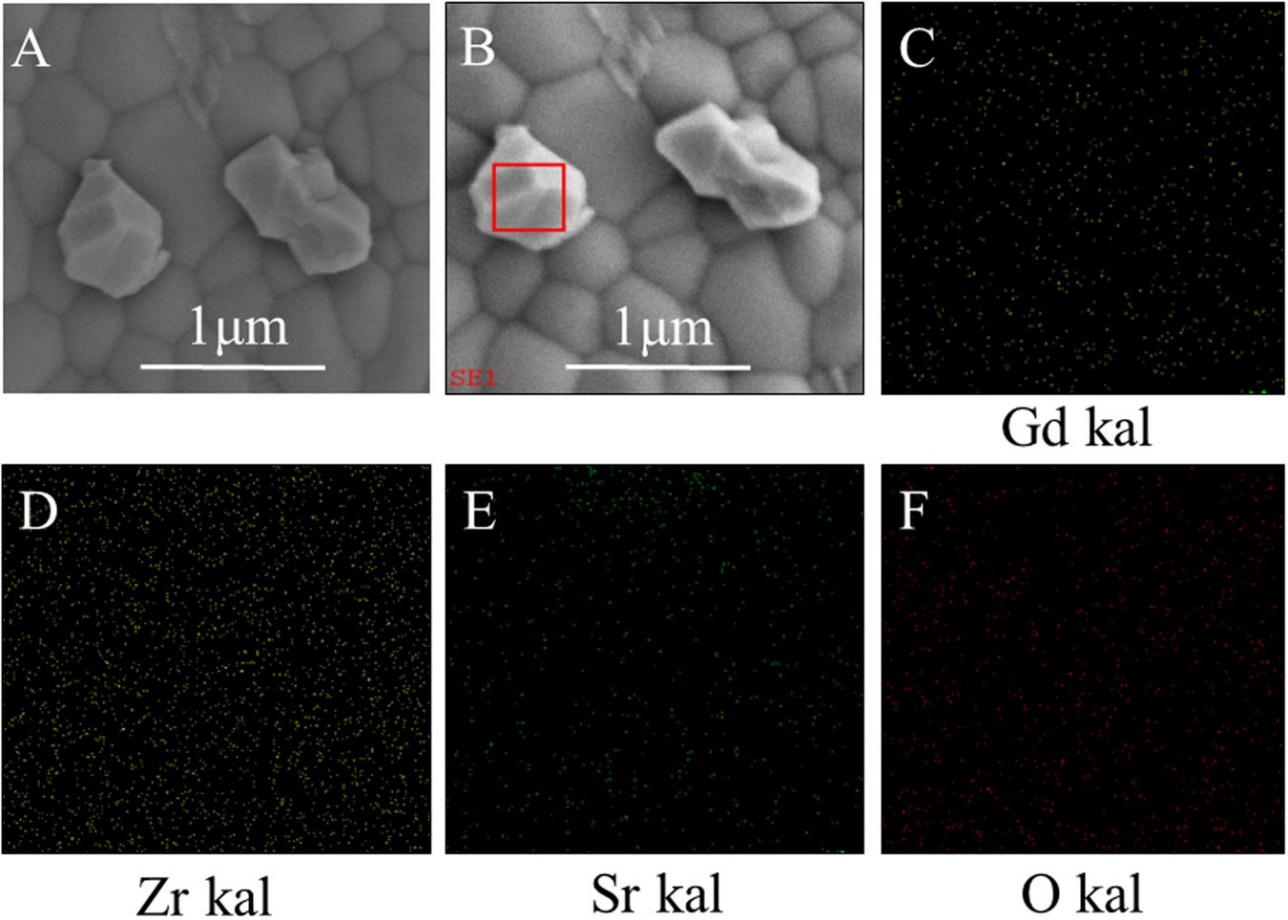
(**A**) SEM photomicrograph of Gd_1.8_Sr_0.2_Zr_2_O_7+*δ*_ (SE image); (**B**) SEM photomicrograph of Gd_1.8_Sr_0.2_Zr_2_O_7+*δ*_ (BSE image); (**C**), (**D**), (**E**), and (**F**): X-ray maps for the constituent ions with the image for Gd_1.8_Sr_0.2_Zr_2_O_7+*δ*_.

The microstructures of Gd_2−x_M_x_Zr_2_O_7+δ_ pellets sintered at 1500 °C for 4 h are given in Fig. 4. As shown, Gd_2−x_M_x_Zr_2_O_7+δ_ substrates prepared by hydrothermal method present the dense pores with a clear boundary during the grains. Pores are seldom found in the grain boundaries. The surface morphology of Gd_2_Zr_2_O_7_ exhibited non-uniform grain structure, high density and homogeneous surface with the grain size in the range of 0.2–1.2 μm. With M^2+^ dopped, the average grain size of the pellets begins to decrease. The average grain size of Gd_1.95_M_0.05_Zr_2_O_7+δ_ (M = Ca, Sr, Ba) pellets is in the range of 0.2–1 μm, 0.1–0.8 μm, and 0.05–0.6 μm, respectively. It should be noted that the Gd_1.95_Ca_0.05_Zr_2_O_7+δ_ pellet exhibits relatively fine grains of 0.2–1 μm in size as shown in Fig. 4B, as compared with other ones of Gd_1.95_Sr_0.05_Zr_2_O_7+δ_ and Gd_1.95_Ba_0.05_Zr_2_O_7+δ_ of which secondary perovskite phase (SrZrO_3_ and BaZrO_3_) seems to appear. To confirm the form of perovskite structure, the BSE image of Gd_1.8_Sr_0.2_Zr_2_O_7+*δ*_ with higher doped concentration for better observation as a good case is shown in Fig. 5. SEM photomicrograph of Gd_1.8_Sr_0.2_Zr_2_O_7+*δ*_ is shown in Fig. 5A. The micrograph manifests heterogeneous grain structures, which could be second phase SrZrO_3_. The BSE image of the same location (Fig. 5B) exhibits high contrast, corresponding to the heterogeneous grain regions of the SE image. To confirm the heterogeneous grain, X-ray mapping was carried out, as shown in Fig. 5C,D,E and F. The heterogeneous grain in the BSE image is found to be rich in gadolinium, zirconium, strontium, and oxygen. The element alanalysis displays that the atomic ratio of the second phase for Sr: Zr: O is close to 1: 1: 3, suggesting that the second phase could be perovskite SrZrO_3_, which is in agreement with the result of XRD. We speculate that the form of perovskite structure CaZrO_3_, SrZrO_3_ and BaZrO_3_ can influence the sensing performance of the sensors based on Gd_1.95_M_0.05_Zr_2_O_7+δ_ substrates. Figure 4C and D shows the SEM photographs of NiO sensitive electrode calcined at 1400 °C for 2 h and the cross-section for porous layer in view of the sensor based on Gd_1.95_Ca_0.05_Zr_2_O_7+δ_ substrates, respectively. It is observed that the surface of NiO SE shows a porous and three-dimensional network structure, which is in favor of prolonging the length of the TPB (NO_2_/NiO/GMZ). This would not only promote the adsorption of NO_2_ to NiO-SE, but capture more electrons to GMZ electrolyte, therefore improving the sensing performance of the sensor.

Figure S2A illuminates the dependence of the conductivities at different operating temperature on the Ca contents of Gd_2−x_Ca_x_Zr_2_O_7+δ_. Clearly, the conductivities increase with increasing temperature when fixing the Ca content. However, the conductivities of Gd_2−x_Ca_x_Zr_2_O_7+δ_ with different Ca doping contents under identical temperature levels are depended on the phase structure. The conductivities increase in the range of pyrochlore phase (0 ≤ x ≤ 0.15) and slightly decline within the fluorite phase (0.2 ≤ x ≤ 0.3) at the same temperature, implying the loss of oxygen vacancy with increasing Ca doping contents. Obviously, the Ca doping increases the mobility of oxide ion of pyrochlore structure due to its unique structural features, resulting in the enhanced conductivity. The highest conductivity of Gd_1.85_Ca_0.15_Zr_2_O_7+*δ*_ reaches up to 9.81 × 10^−3^ S cm^−1^ at 1173 K. Very good linear relation of the Arrhenius plots of grain conductivity of the Gd_2−x_Ca_x_Zr_2_O_7+*δ*_ electrodes in Fig. S2B indicates that the migration of oxide ions in the series is thermally activated. The relationship between the activation energy E_a_ calculated from the slope in the Arrhenius plots and the Ca contents in Fig. S2C displays that E_a_ gradually declines in the range of pyrochlore phase and gradually increases within the fluorite phase with increasing the Ca content, suggesting that the addition of Ca plays an important role in the E_a_. The minimum E_a_ value is 0.36 eV that happens on Gd_1.85_Ca_0.15_Zr_2_O_7+*δ*_. The low E_a_ would facilitate the oxide-ion hopping, which spontaneously results in an increase in conductivity.

**Figure 6:**
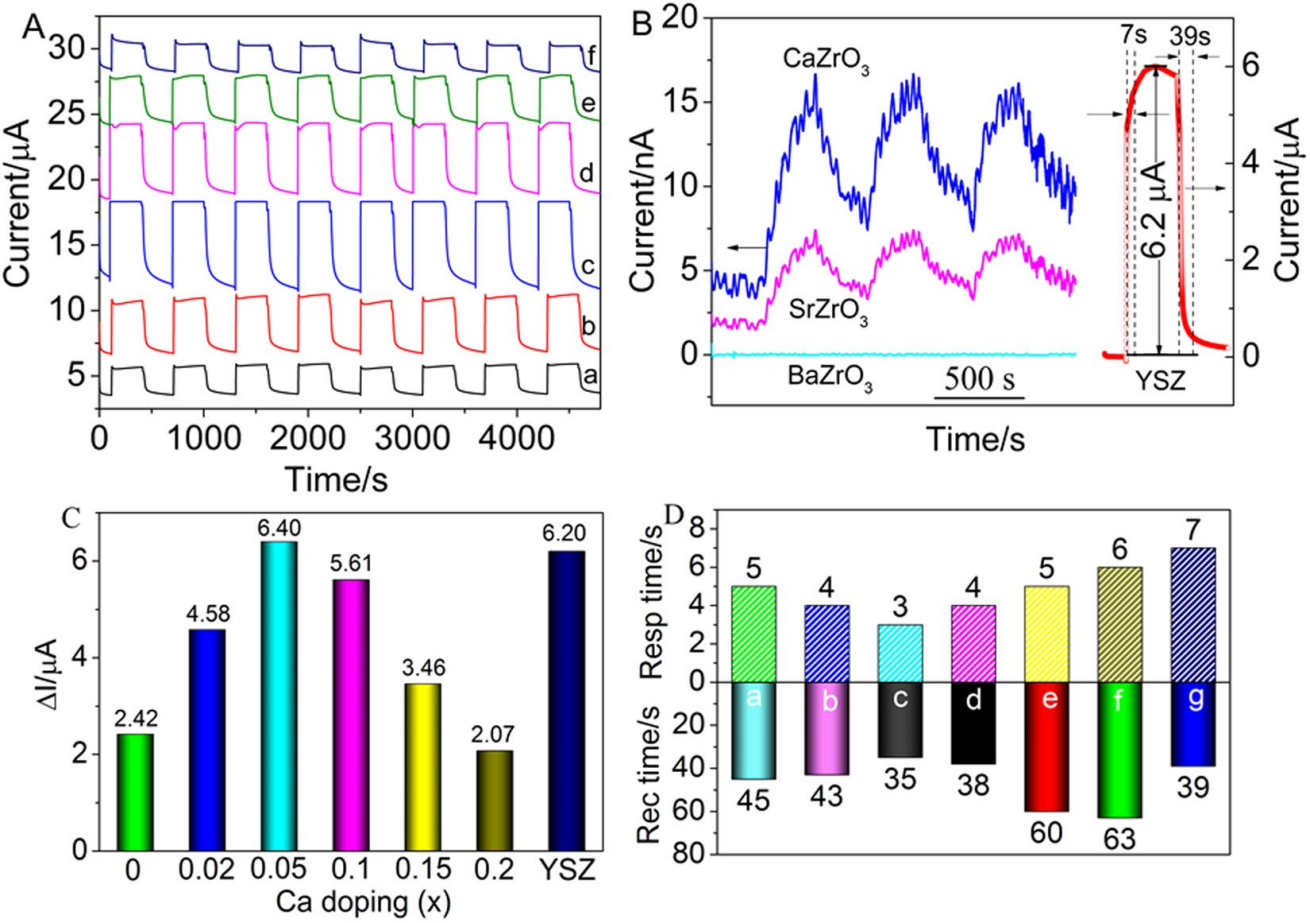
(**A**) Response transients of the sensor based on Gd_2−*x*_Ca_*x*_Zr_2_O_7+*δ*_ substrates to 500 ppm NO_2_ in the presence of 5 vol. % O_2_ at 500 °C (applied potential −300 mV, flow rate 200 cm^3^/min): (a) x = 0, (b) x = 0.02, (c) x = 0.05, (d) x = 0.1, (e) x = 0.15, (f) x = 0.2; (**B**) response transients of the sensors based on CaZrO_3_, SrZrO_3_, BaZrO_3_, and YSZ substrates to 500 ppm NO_2_ in the presence of 5 vol. % O_2_ at 500 °C (applied potential −300 mV, flow rate 200 cm^3^/min); (**C**) the effect of calcium doping content on the response signal ΔI in 500 ppm NO_2_ at 500 °C; (**D**) the relationship between response time, recovery time and Ca doping concentration of the sensor based on Gd_2−*x*_Ca_*x*_Zr_2_O_7+*δ*_ and YSZ substrates to 500 ppm NO_2_: (a) x = 0, (b) x = 0.02, (c) x = 0.05, (d) x = 0.1, (e) x = 0.15, (f) x = 0.2, (g) YSZ.

The amperometric response and recovery transients of the sensors based on Gd_2−x_Ca_x_Zr_2_O_7+δ_ substrates when exposed to 500 ppm NO_2_ with a polarized potential of −300 mV at 500 °C are shown in Fig. 6A, where in order to better distinguish the response curve, the base current levels have been shifted. Clearly, the response signals rapidly increase upon injecting the NO_2_ gas and sharply recover to an original level after removal of the NO_2_ gas. In our study, the response current value was defined as the difference of current value between the sample gas and base gas (*ΔI* = |*I*_*gas*_ − *I*_*base*_|, where *I*_*gas*_ and *I*_*base*_ referred to the response current values in the targeted concentration and 0 ppm NO_2_). As seen from Fig. 6C, the ΔI value of the undoped Gd_2_Zr_2_O_7_ is relatively small (2.42 μA) at 500 °C. After introducing Ca ions, ΔI reaches the maximum 6.40 μA for *x* = 0.05 at 500 °C as compared with the sensor based on YSZ (6.20 μA) commercially used. However, ΔI of *x* = 0.1, 0.15, 0.2 drops to 5.61, 3.46, and 2.07 μA, respectively, suggesting that the calcium doping concentrations have a great effect on the ΔI values of the sensor at 500 °C. It is reasonable that the higher the conductivity is, the better the sensing performance is. However, the sensor based on Gd_1.95_Ca_0.05_Zr_2_O_7-δ_ substrate gives the highest ΔI value rather than Gd_1.85_Ca_0.15_Zr_2_O_7-δ_ with the highest conductivity. The sensing performance of the sensor is related to many factors as the degree of NO_2_ enrichment at the interface between SE and solid electrolyte is diverse. Another reason is mainly because the excessive Ca ions combine with Zr ions to form to a perovskite structure CaZrO_3_, which can be inert for NO_2_ detection. To confirm the NO_2_ sensing performance, the amperometric response and recovery transients of the sensors based on CaZrO_3_, SrZrO_3_ and BaZrO_3_ substrates when exposed to 500 ppm NO_2_ with a polarized potential of −300 mV at 500 °C are shown in Fig. 6B. Clearly, the ΔI values of the sensors are very low and basically ignore. It was reported that CaZrO_3_, SrZrO_3_ and BaZrO_3_ belong to proton conductivities^41–43^, which results in not only the loss of oxygen transport function, but the decrease of NO_2_ adsorption capacity, which restrains the electrode catalytic reaction of Eq. 3. Consequently, the ΔI values of the NO_2_ sensors will be lowered. The response and recovery time shown in Fig. 6D confirms the conclusion. The response time that is commonly defined as the time that the resistance of the sensor reaches to 90% of the saturation value when the sensor is exposed to NO_2_ for x = 0, 0.02, 0.05, 0.1, 0.15 and 0.2 is 5, 4, 3, 4, 5 and 6 s, respectively. The recovery time that is in general defined as the time required for recovering the 90% of the original resistance for x = 0, 0.02, 0.05 and 0.1 is 45, 43, 35 and 38 s, respectively, whereas the recovery time is obviously delayed and exceeds 60 s when Ca content is greater than 0.1. This can be ascribed to more CaZrO_3_ produced as Ca ions incerese, which makes the sensitivity lower. The results in this work indicate that among the sensors based on Gd_2−x_Ca_x_Zr_2_O_7+*δ*_ substrates, the sensor based on Gd_1.95_Ca_0.05_Zr_2_O_7+*δ*_ substrate displays the optimal device with the highest ΔI (6.4 μA), the shortest response (3 s) and recovery time (35 s), which is obviously better than the sensor based on commercial YSZ with ΔI (6.2 μA), the response (7 s) and recovery time (39 s).

**Figure 7:**
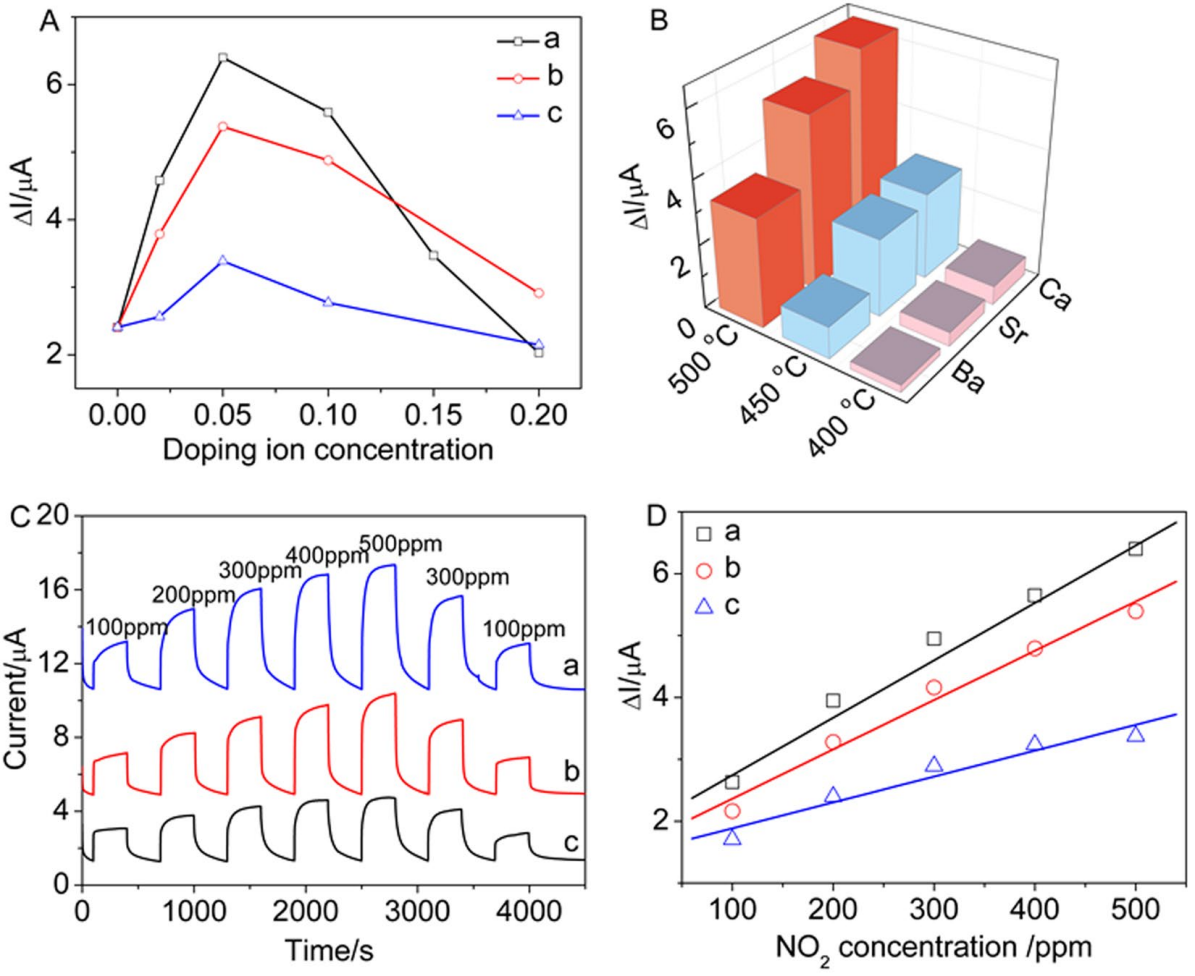
(**A**) The effect of doping concentration for different element on the ΔI value in 500 ppm NO_2_ at 500 °C: (a) Gd_2−x_Ca_x_Zr_2_O_7+*δ*_, (b) Gd_2−x_Sr_x_Zr_2_O_7+*δ*_, (c) Gd_2−x_Ba_x_Zr_2_O_7+*δ*_; (**B**) the effect of doping element and temperature on response signal ΔI in 500 ppm NO_2_ at 500 °C; (**C**) Amperometric response and recovery transients to various NO_2_ concentrations of the sensor in the presence of 5 vol. % O_2_ at 500 °C (applied potential −300 mV, flow rate 200 cm^3^/min): (a) Gd_1.95_Ca_0.05_Zr_2_O_7+δ_, (b) Gd_1.95_Sr_0.05_Zr_2_O_7+δ_, (c) Gd_1.95_Ba_0.05_Zr_2_O_7+δ_; (**D**) the relationship between the response current values ΔI and NO_2_ concentrations at 500 °C: (a) Gd_1.95_Ca_0.05_Zr_2_O_7+δ_, (b) Gd_1.95_Sr_0.05_Zr_2_O_7+δ_, (c) Gd_1.95_Ba_0.05_Zr_2_O_7+δ_.

For the purpose in comparison of the sensing performances, the effect of different doping element (Ca, Sr and Ba) and doping concentration on the ΔI values in 500 ppm NO_2_ at 500 °C is shows in Fig. 7A. Obviously, the ΔI values of the sensors based on Gd_2−x_Ca_x_Zr_2_O_7+*δ*_ substrates are higher than those for Gd_2−x_Sr_x_Zr_2_O_7+*δ*_ and Gd_2−x_Ba_x_Zr_2_O_7+*δ*_ substrates. This is mainly because SrZrO_3_ and BaZrO_3_ are easy to be produced even if the doping concentration is very low such as x = 0.02 for Gd_2−x_Sr_x_Zr_2_O_7+*δ*_ and Gd_2−x_Ba_x_Zr_2_O_7+*δ*_, as seen from XRD results above. Thus the effect of Sr and Ba doping on the sensing performance is weak. For each doping element, the sensors based on the substrates for x = 0.05 manifest the highest ΔI value. It is concluded that the pyrochlore-phase Gd_1.95_M_0.05_Zr_2_O_7+δ_ is a kind of outstanding electrolyte for NO_2_ sensor. Therefore, the effect of different operating temperature on the ΔI values of the sensors based on Gd_1.95_M_0.05_Zr_2_O_7+*δ*_ substrates in 500 ppm NO_2_ is presented in Fig. 7B. Clearly, when increasing operating temperature at a fixed doping element, the ΔI values of the sensors increase as well. For example, the ΔI value is 0.59 μA for the sensor based on Gd_1.95_Ca_0.05_Zr_2_O_7+*δ*_ substrate at 400 °C. With increasing temperature such as 500 °C, the ΔI value increases to 6.4 μA. This is mainly attributed to the enhanced conductivity and electrochemical reaction rate when increasing temperature. In addition, over the whole range of temperature, the sensor based on Gd_1.95_Ca_0.05_Zr_2_O_7-δ_ substrate gives the highest ΔI value of 6.4 μA, exhibiting highly sensing performance to NO_2_ at the bias potential of −300 mV at 500 °C.

To investigate in depth the sensing performance, the dynamic amperometric response and recovery transients for the sensors based on Gd_1.95_M_0.05_Zr_2_O_7+*δ*_ substrates as a function of the NO_2_ concentration at the applied potential of −300 mV at 500 °C are presented in Fig. 7C. The response current value gradually increases when NO_2_ concentration increases from 100 to 500 ppm. In the case of the sensor based on Gd_1.95_Ca_0.05_Zr_2_O_7+*δ*_ substrate, the ΔI value of the sensor is 2.63 μA for 100 ppm NO_2_ at 500 °C. When NO_2_ concentration ascends to 500 ppm, the response current value raises to 6.40 μA at under the same conditions. Very good linear relationships between the response signal Δ*I* and NO_2_ concentrations in the range from 100 to 500 ppm are achieved (Fig. 7D), indicating that the sensors based on Gd_1.95_M_0.05_Zr_2_O_7+*δ*_ substrates have an excellent sensitive performance to NO_2_ at 500 °C. The sensitivity of the sensor is defined as the slope of response current value ΔI on the target gas concentration at a certain temperature, which can be calculated from the fitting results of ΔI on various NO_2_ concentrations. And the sensitivities of the Gd_1.95_Ca_0.05_Zr_2_O_7+*δ*_, Gd_1.95_Sr_0.05_Zr_2_O_7+*δ*,_ Gd_1.95_Ba_0.05_Zr_2_O_7+*δ*_ based on sensor is 9.28, 7.97, and 4.18 nA/ppm at 500 °C, respectively. It is manifested that Gd_1.95_Ca_0.05_Zr_2_O_7+δ_ substrate is most excellent substrate among Gd_2−x_M_x_Zr_2_O_7+δ_ ones of the sensor. Therefore, the dynamic amperometric response and recovery transients to NO_2_ in concentrations range of 100–500 ppm for the sensor based on Gd_1.95_Ca_0.05_Zr_2_O_7+*δ*_ substrate with a polarized potential of −300 mV at 400, 450 and 500 °C are investigated and presented in Fig. S3A. The response current value is almost linear to the NO_2_ concentration from 100 to 500 ppm at 400, 450 and 500 °C (Fig. S3B). It is found that the response signal is very low at 400 °C, whereas increasing operating temperature, the response signal greatly increases at each NO_2_ concentration. The sensor based on Gd_1.95_Ca_0.05_Zr_2_O_7+*δ*_ substrate exhibits the highest ΔI value of 6.40 μA with 500 ppm NO_2_ at 500 °C. For NO_2_ sensor, the response current depends on the electrochemical catalytic activities of the NiO sensing electrode at TPB. The number of NO_2_ molecules adsorbed on the sensing electrode increases when NO_2_ concentration changes from 100 to 500 ppm, implying that more oxygen ions (O^2−^) would be produced through the cathodic reaction of Eq. (3). As a result, the response current value of the sensor is enhanced. While the electrochemical reaction rate of both Eqs (3) and (5) increases when increasing operating temperature at a fixed NO_2_ concentration, causing the response current value of the sensor to increase as well. The sensitivities fitted from Fig. S3B are 0.97, 3.59, and 9.28 nA/ppm at 400, 450 and 500 °C, respectively. It is found that the sensitivity greatly increases as increasing operating temperature. In practical automobile exhaust application, the concentration for NO_2_ gas detection can be very low. Therefore, the response and recovery transients of the Gd_1.95_Ca_0.05_Zr_2_O_7+*δ*_ based sensor towards 25–500 ppm NO_2_ with lower NO_2_ concentration at 500 °C is exhibited in Fig. S3C. Figure S3D depicts the good liner fitting results of ΔI values and NO_2_ concentrations in the range from 25 to 500 ppm. The great linear correlations are beneficial to the practical gas sensing application.

**Figure 8:**
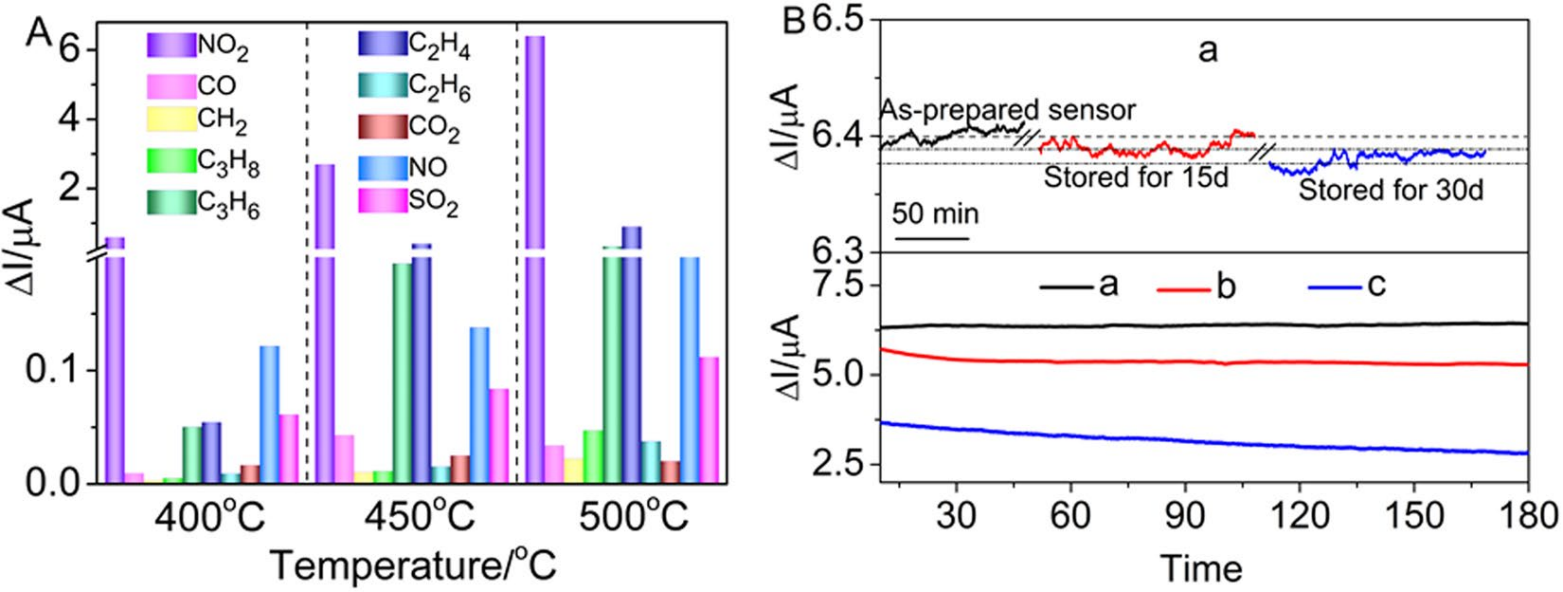
(**A**) Selectivity of the sensor based on Gd_1.95_Ca_0.05_Zr_2_O_7+*δ*_ substrate in 500 ppm various gases at 400, 450 and 500 °C, respectively (applied potential −300 mV, flow rate 200 cm^3^/min); (**B**) Stability test for the sensors at 500 °C in the presence of 500 ppm NO_2_ (applied potential −300 mV, flow rate 200 cm^3^/min): (a) Gd_1.95_Ca_0.05_Zr_2_O_7+*δ*_; (b) Gd_1.95_Sr_0.05_Zr_2_O_7+*δ*_; Gd_1.95_Ba_0.05_Zr_2_O_7+*δ*_ substrate.

Actual automobile exhaust pollutant might include various coexist gas, so it is necessary for us to evaluate the NO_2_ sensing performance in more variable conditions with other coexist gas. The cross-sensitivities to various gases for the sensor based on Gd_1.95_Ca_0.05_Zr_2_O_7+δ_ substrate at 400, 450, and 500 °C is exhibited in Fig. 8A. It is observed that the present sensor displayed an excellent sensitivity and selectivity for NO_2_ over the other gases tested, while a slight cross sensitivity was detected with compounds such as CO, CH_4_, C_3_H_8_, C_3_H_6_, NO, SO_2_, C_2_H_4_, CO_2_ and C_2_H_6_. The maximum response current reached 6.4 μA towards 500 ppm NO_2_ at 500 °C, outdistancing the other gases. Compared with NO_2_ gas, the ΔI value of interference gases such as CO, CH_4_, C_3_H_8_, C_3_H_6_, NO, SO_2_, C_2_H_4_, CO_2_ and C_2_H_6_ is fairly small and basically ignored in whole of operating temperatures.

The stability of the sensors based on Gd_1.95_Ca_0.05_Zr_2_O_7+δ_, Gd_1.95_Sr_0.05_Zr_2_O_7+δ_ and Gd_1.95_Ba_0.05_Zr_2_O_7+δ_ substrates was measured for 3 h upon exposure to 500 ppm NO_2_ gas at 500 °C, as shown in Fig. 8B. The ΔI value of the sensor based on Gd_1.95_Ca_0.05_Zr_2_O_7+δ_ basically maintains constant and slightly decreases on Gd_1.95_Sr_0.05_Zr_2_O_7+δ_, whereas the ΔI value of the sensor based on Gd_1.95_Ba_0.05_Zr_2_O_7+δ_ obviously decreases during whole the test, suggesting excellent stability towards Gd_1.95_Ca_0.05_Zr_2_O_7+δ_ substrate as compared to Gd_1.95_Sr_0.05_Zr_2_O_7+δ_ and Gd_1.95_Ba_0.05_Zr_2_O_7+δ_ ones. Meantime, good NO_2_ sensors should possess the ability to maintain a reliable stabilized sensing performance after a period of storage. The ΔI values with slight fluctuation decreases by 0.01 and 0.02 μA, which only accounts for 0.16 and 0.31% of the original response current value 6.4 μA after the sensors based on Gd_1.95_Ca_0.05_Zr_2_O_7+δ_ was stored for half a month and a month, respectively, indicating good long-term stability for NO_2_ detection.

The coordination between the GMZ electrolyte and the SE can be one of pivotal factors of the NO_2_ sensing performance. When the negative electrode is applied on SE, the SE preferentially absorbs NO_2_ molecules on the surface other than O_2_ or other rest of gas among atmosphere as the electron affinity of NO_2_ is about five times higher than that of oxygen^44^. NO_2_ gas diffuses through the porous NiO along TPB to GMZ electrolyte due to its large adsorption capacity at mild temperature, which necessarily extends the length of TPB. This makes NO_2_ gas fewer contacts with the surface of the NiO grains and reach TPB interface without serious catalytic decomposition of NO_2_. The mass spectrum trace signal of off-gas (500 ppm NO_2_ + 5% O_2_ + He) of the sensor based on Gd_1.95_Ca_0.05_Zr_2_O_7+δ_ substrate at 500 °C in Fig. S4 affirms the conclusion. According to Fig. S4, NO and O_2_ might be resultant gases after the sensing behavior happened at the electrochemical reaction, and the changes of other NO_x_ are too small to ignore. The content of N_2_ that is obtained from NO_2_ gas decomposition into N_2_ on SE and NO reduction to N_2_ at the cathode is very low, indicating high sensitivity for NO_2_ detection.

## Conclusions

A highly-stable amperometric-type NO_2_ sensor based on pyrochlore-phase Gd_2−x_M_x_Zr_2_O_7+δ_ solid electrolyte with NiO as the SE and a noble metal Pt as the RE was fabricated and investigated here. The sensor presented excellent sensing performance to NO_2_ gas. The response current value at −300 mV was almost linear to NO_2_ concentration in the range of 0~500 ppm at 400–500 °C. The optimal sensor based on the Gd_1.95_Ca_0.05_Zr_2_O_7+δ_ substrate gave the highest NO_2_ sensitivity (9.28 nA/ppm), the maximum response current value (6.4 μA), and the shortest 90% response (3 s) and 90% recover (35 s) time to 500 ppm NO_2_ at 500 °C, which is better than that of commercial YSZ under the same condition. The outstanding selectivity and high stability towards NO_*2*_ sensing of the sensors based on Gd_2−x_M_x_Zr_2_O_7+δ_ are expected to a promising application in monitoring exhaust emission of motor vehicles.

## Methods

### Preparation of GMZ electrolyte

The pyrochlore-phase Gd_2−x_M_x_Zr_2_O_7-δ_ (GMZ, M = Ca, Sr, and Ba, x = 0–0.3) oxides were synthesized through a urea hydrolysis-based hydrothermal method. The stoichiometric amount of Gd(NO_3_)_3_·6H_2_O (99.99% purity), M(NO_3_)_2_ (M = Ca, Sr, and Ba) (AR Grade), and ZrOCl_2_·8H_2_O (AR Grade) were first dissolved in deionized water, and the total cation concentration was fixed at 0.25 mol/L. Then urea (AR Grade) as precipitation agent was added to the reaction solutions above with the molar ratio of the total cation: urea = 1: 2.5. Thereafter, 80 mL solution was poured into a Teflon bottle (inner volume: 100 mL), which was kept in a stainless steel autoclave. After the autoclave was sealed tightly, it was removed into an oven with controlling temperature to hydrothermal treatment at 180 °C for 24 h. After hydrothermal treatment, white precipitates were centrifugally separated, washed with deionized water for three times, and subsequently dried at 110 °C for 24 h in air. The as-prepared Gd_2−x_M_x_Zr_2_O_7-δ_ powders were calcined in still air at 600 °C for 4 h.

### Sensor fabrication and characterization

The samples calcined at 600 °C above were uniaxially pressed into a pellet (8 mm diameter, 2 mm thickness). Subsequently, the molded pellet was further compacted by cold isostatic pressing at 280 MPa for 5 min. Finally, the compacts were sintered at 1500 °C for 4 h in air. The NiO paste was painted on one of the surfaces of GMZ pellet by screen printing technique and then sintered at 1400 °C for 2 h to create the sensing electrode. Pt paste was painted on the back-side of the electrolyte, and then two Pt wires (0.2 mm diameter) were wound around the NiO and Pt surfaces to make contact with the sensor, respectively. Then the samples were calcined at 1000 °C for 1 h in air to get the (Pt) NiO/GMZ/Pt sensor. Phase analysis was done on a Panalytical X’Pert Pro diffractometer at 40 kV and 40 mA with a step size of 0.0167° at a scanning rate of 4° min^−1^, using Co K_α_ radiation and then revised by Cu K_α_. Scanning electron microscopy (SEM, HitachiS4800 instrument) was applied for observing the morphology of the samples. The Raman spectra were measured on a multichannel modular triple Raman system (inVia Reflex, Renishaw Corp.) with confocal microscopy at room temperature excited with the 532 nm line of an Ar laser. The complex-impedance measurements of the GMZ electrolytes were carried out in ambient air, and typically in the frequency range of 1 MHz to 0.01 Hz with signal amplitude of 5 mV using the Zahner IM6 electrochemical workstation.

### Evaluation of sensing properties

NO_2_ sensing properties were carried out on a fixed bed continuous flow reactor. The fabricated sensors were held in a quartz glass (i. d. 10.0 mm) with heating tube furnace in the temperature range 400–500 °C. The gas environment consisted of a changing concentration of NO_2_ (0–500 ppm) with base gases (5 vol. % O_2_ + N_2_ balance) at a total flow rate of 200 mL/min, which was controlled by mass flow meter. The amperometric responses of the sensors were measured by potentiostatic method at −300 mV using the electrochemical work station (Instrument corporation of Shanghai, China, CHI600E). The trace signal of off-gas of the sensor placed in testing tube was performed on a mass spectrometry (Dycor Dymaxion, DME200MS) with Pt wires connected to CHI600E electrochemical workstation at the applied potential −300 mV and the flow rate of 200 mL/min. The testing gas (500 ppm NO_2_/He + 5 vol. % O_2_/He + He balance) and base gas (5 vol. % O_2_/He + He balance) were used to avoid interfering by N_2_ in normal mixed gas.

## Electronic supplementary material

Supporting information
