# Supplementary material for: Alkaline-Earth Metals-Doped Pyrochlore Gd2Zr2O7 as Oxygen Conductors for Improved NO2 Sensing Performance
Source: Sci Rep. 2017 Jul 5;7:4684. doi: 10.1038/s41598-017-04920-1 (PMC5498611; doi:10.1038/s41598-017-04920-1)
Supplement: Supplementary file 1 — Supporting information [file 41598_2017_4920_MOESM1_ESM.pdf]

Supplementary Information

Alkaline-Earth Metals-Doped Pyrochlore $Gd_2Zr_2O_7$ as Oxygen Conductors for Improved NO_2 Sensing Performance

Fulan Zhong*[a], Jiwu Zhao[a], Lanqian Shi[a], Yihong Xiao[a], Guohui Cai[a], Yong Zheng[a] & Jinlin Long*[b]

[a]National Engineering Research Center of Chemical Fertilizer Catalyst (NERC-CFC), School of Chemical Engineering, Fuzhou University, Gongye Road No.523, Fuzhou 350002, Fujian, P. R. China

[b]State Key Laboratory of Photocatalysis on Energy and Environment, School of Chemistry, Fuzhou University, Fuzhou 350116, P. R. China

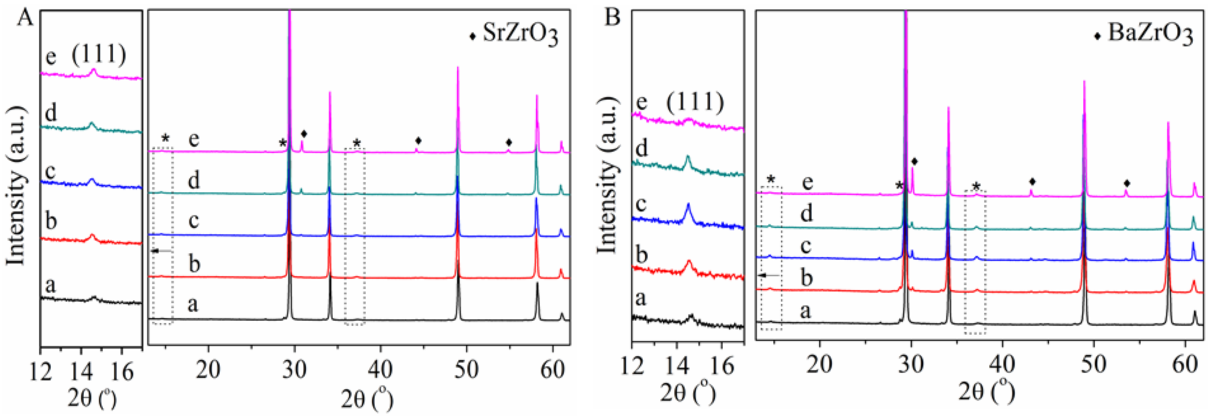

Fig. S1. XRD patterns of $Gd_{2-x}Sr_xZr_2O_{7+\delta}$ (A) and $Gd_{2-x}Ba_xZr_2O_{7+\delta}$ (B) powders calcined at 1500 ^oC for 4 h: (a) x=0, (b) x=0.02, (c) x=0.05, (d) x=0.1, (e) x=0.2.

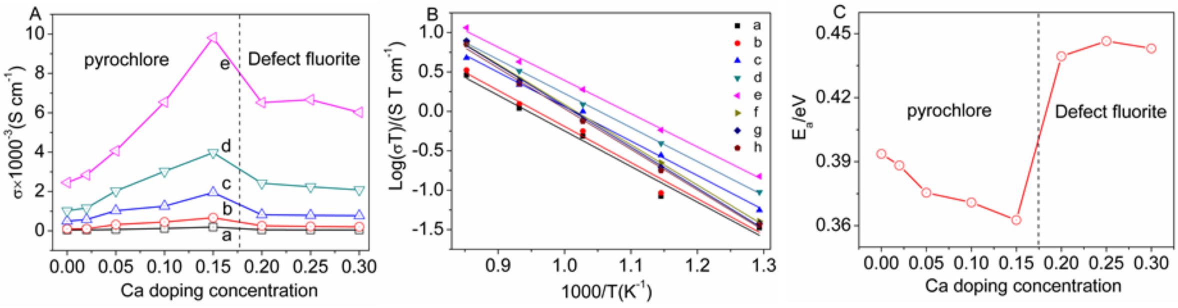

Fig. S2. (A) The dependence of the conductivity at different operating temperature on the Ca contents of $Gd_{2-x}Ca_xZr_2O_{7+\delta}$: (a) 500 °C, (b) 600 °C, (c) 700 °C, (d) 800 °C, (e) 900 °C; (B)Arrhenius plots of grain conductivity of $Gd_{2-x}Ca_xZr_2O_{7+\delta}$ measured in air: (a) x=0, (b) x=0.02, (c) x=0.05, (d) x=0.1, (e) x=0.15, (f) x=0.2, (g) x=0.25, (h) x=0.3; (C) The relationship between the activation energy E_a calculated from the slope in the Arrhenius plots and the Ca contents.

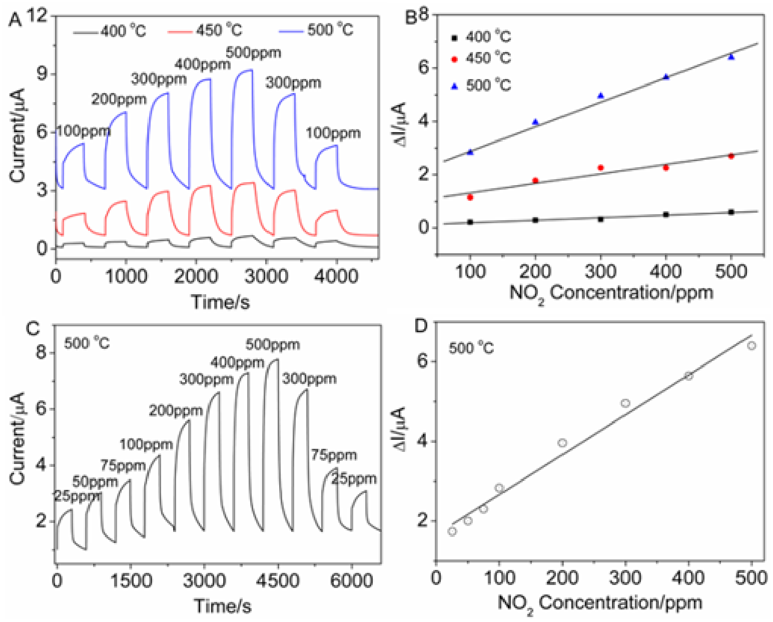

Fig. S3. (A) Amperometric response and recovery transients to various NO_2 concentrations of the sensor based on $Gd_2Ca_{0.05}Zr_2O_{7+\delta}$ substrates in the presence of 5 vol. % O_2 (applied potential –300 mV, flow rate 200 cm^3/min) at 400 ^oC, 450 ^oC, and 500^oC; (B) the relationship between the response current values ΔI and NO_2 concentrations of the sensor based on $Gd_2Ca_{0.05}Zr_2O_{7+\delta}$ substrates at 400, 450, and 500^oC. (C) Amperometric response and recovery transients to 25-500 ppm NO_2 of the sensor based on $Gd_2Ca_{0.05}Zr_2O_{7+\delta}$ substrates at 500^oC; (D) the relationship between the response current values ΔI and NO_2 concentrations of the sensor based on $Gd_2Ca_{0.05}Zr_2O_{7+\delta}$ substrates at 500^oC.

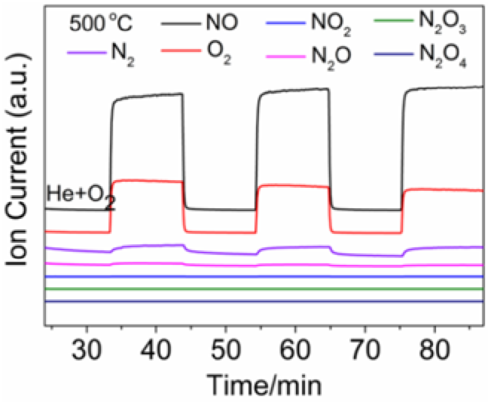

Fig. S4. The mass spectrum trace signal of off-gas (500 ppm NO_2 + 5 vol. % O_2 + He) of the sensor based on $Gd_{1.95}Ca_{0.05}Zr_2O_{7+\delta}$ substrate at 500 ^{o}C.
